# Multidrug resistance in the standardized treatment of colon cancer harboring a rare fibrosarcoma B-type (BRAF) p.N581I mutation: a case report

**DOI:** 10.3389/fonc.2023.1175693

**Published:** 2023-07-14

**Authors:** Xiaoyan Wang, Chenyi Zhao, Yang Gong, Ying Wang, Feng Guo

**Affiliations:** Department of Oncology, Suzhou Municipal Hospital, The Affiliated Suzhou Hospital of Nanjing Medical University, Suzhou, China

**Keywords:** BRAF p.N581I, colorectal cancer metastasis, targeted therapy, immunotherapy, multidrug resistance, anti-EGFR monoclonal antibody

## Abstract

BRAF non-V600 mutations are a distinct molecular subset of colorectal cancer (CRC) that has little to no clinical similarity to the BRAF V600 mutations. It is generally considered that the BRAF non-V600 mutations correlate with better survival of CRC patients. In this report, we present an unusual case of that a midlife female patient who was initially diagnosed with stage IIIC colon cancer, and multiple metastases were found 25 months after radical surgery. Next-generation sequencing (NGS) revealed the BRAF p.N581I (c.1742A>T) mutation. She received chemotherapy, targeted therapy, and immunotherapy. However, the disease progressed rapidly with rare metastasis of the bone and cerebellum. This case highlights that the BRAF non-V600 mutations, such as BRAF p.N581I mutant, may lead to resistance to epidermal growth factor receptor (EGFR) inhibitors and result in a rapid course in colorectal cancer. The role of BRAF p.N581I mutation in colorectal cancer demands more attention.

## Introduction

CRC is the third most diagnosed cancer in the world, and the main cause of death is metastases (1). CRC can be classified into different subtypes, which are characterized by specific molecular and morphological alterations (2). Among those, the activation of the mitogen-activated protein kinase (MAPK) signaling pathway is one of the most important pathways involved in the occurrence of CRC, mainly including Kirsten rat sarcoma viral oncogene homologue (KRAS), V-RAF murine sarcoma viral oncogene homologue B1 (BRAF), and mitogen-activated protein (MEK) (3). The BRAF mutations occur in approximately 10% of CRC patients, most of them at the V600 amino acid, a T1799A transversion in exon 15. However, the small number of patients with the BRAF non-V600 mutations limits the further understanding of these particular types of mutations. The clinical associations, genetic interactions, and therapeutic implications of the BRAF non-V600 mutations have not been explored comprehensively yet (4, 5). This report presents a Chinese woman with rapidly progressive metastatic colon cancer, who had a somatic BRAF p.N581I mutation and was resistant to EGFR-inhibition combined with chemotherapy, causing an unfavored progression. The rapid progression was beyond our cognition of BRAF non-V600 mutations from the previous studies. Here, we report this case and discuss the current research on the BRAF non-V600 mutations in colorectal cancer.

## Case presentation

**Figure 1:**
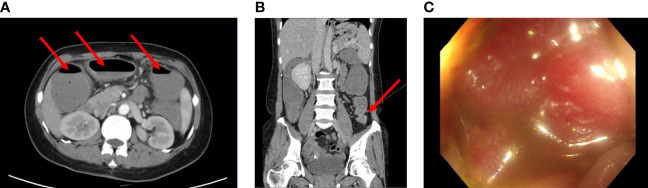
**(A, B)** Computed tomography images of the patient before surgery (2018-11). Enhanced CT scans of the abdomen revealed the dilatation and gas-fluid level of the colon. **(C)** Electronic colonoscopy prompts a large cauliflower lump in the colon, situated approximately 70cm from the anal verge. The lesion completely occluded the colon lumen.

**Figure 2:**
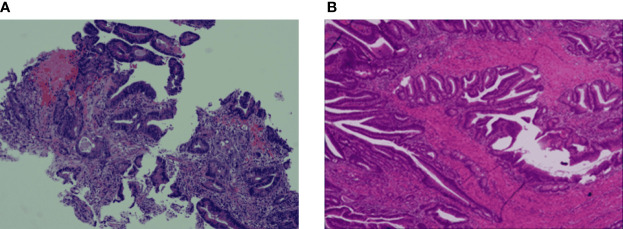
Haematoxylin-eosin staining (H&E) and IHC staining of the colon cancer tissue specimens. **(A)** The colon biopsy result is low differentiated adenocarcinoma, partially mucinous adenocarcinoma. **(B)** The tumour infiltrates the whole intestinal wall into extraneous fibrous adipose tissue.

**Figure 3:**
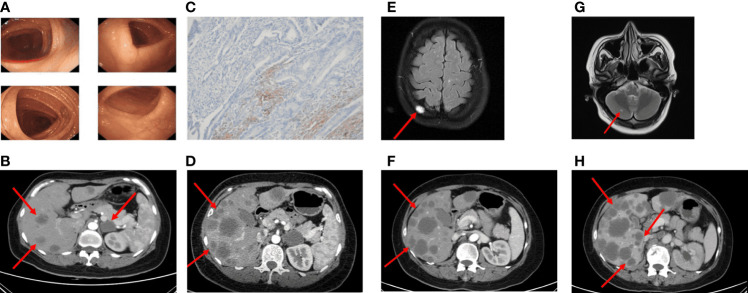
Imaging in the palliative care process CT revealed metastatic lesions. **(A)** Colonoscopy found no abnormal changes (2020-08). **(B)** Imaging examinations performed more than 2 years after surgery (2021-02). Multiple metastatic lesions in the liver. **(C)** The PD-L1 IHC suggested the PD-L1 expression was 2%. **(D)** After four cycles of FOLFIRI plus cetuximab treatment, CT scans revealed significant enlargement of liver metastasis (2021-04). **(E)** MRI scans after five cycles of FOLFIRI plus bevacizumab showed the new abnormal high density shadow of the right skull, considering metastasis (2021-06). **(F, G)** After two months of the therapy of mFOLFOX6 and palliative radiation, hepatic metastatic lesions on CT were similar to the previous, the cranial MRI suggested cerebellar metastasis (2021-09). **(H)** The patient's last CT reexamination showed liver metastasis progressed again (2021-12).

A 41-year-old woman was admitted to our hospital with complaints of abdominal pain and abdominal distension with anal stop defecation and exhaust for 5 days on 27 November 2018. Enhanced computed tomography (CT) of the abdomen revealed colon dilatation, hydrops, and gas-fluid level, considering the possibility of bowel obstruction (**Figures 1A, B**). An electronic colonoscopy revealed a large cauliflower lump in the colon, situated approximately 70 cm from the anal verge (**Figure 1C**). The lesion completely occluded the colon lumen, and the endoscope could not pass through. Rapid pathological detection revealed an adenocarcinoma. Carcinoembryonic antigen (CEA) was 15.77 ng/mol. The patient underwent total resection of the sigmoid flexure cancer on 24 December 2018. Postoperative pathology showed a poorly differentiated adenocarcinoma in the sigmoid flexure (pT4aN2M0, **Figure 2A**). The diameter of the tumor was approximately 4 cm. The tumor infiltrated the entire intestinal wall and accumulated extra-serosal fibrous adipose tissues. Meanwhile, eight mesenteric lymph nodes metastases were observed. Immunohistochemistry (IHC) results were as follows (**Figure 2B**): microsatellite stable/mismatch repair-proficient (MSS/pMMR), MLH1 (+), MSH2 (+), MSH6 (+), PSM2 (+), KI67 (60%), CDX2 (+), ER (−), and Vimentin (−). Therefore, six cycles of adjuvant chemotherapy (XELOX: oxaliplatin, 130 mg/m^2^ on day 1; capecitabine, 1,000 mg/m^2^ twice daily on days 1–14, orally) strategy was recommended from 12 December 2018 to 10 April 2019. After postoperative adjuvant chemotherapy, the patient was followed up in the outpatient clinic, and a CT scan was performed every 6 months. The physical examination and vital signs were within normal limits for more than 1 year. The last colonoscopy was performed in August 2020, with no anomaly either (**Figure 3A**).

Until February 2021, the re-examination of the abdomen CT suggested multiple hepatic metastases, and the adrenal glands also metastasize, accompanied by the retroperitoneal lymph nodes enlargement (**Figure 3B**). The CT evaluations were progressive disease (PD). Then, the patient received a first-line course of palliative chemotherapy with FOLFIRI (irinotecan 240 mg day 1, leucovorin 500 mg day 1, 5-FU 500 mg IV bolus on day 1, then 3,000 mg 46 h IV continuous infusion, every 2 weeks). The NGS analysis was performed from formalin-fixed and paraffin embedded (FFPE) and blood-based circulating tumor DNA (ctDNA, taken in February 2021). The mutant genes and mutation abundance examined from the FFPE sample were as followed: TP53 p.E298* (37.52%), APC p.Q1429* (33.35%), RAF1 p.R391W (23.56%), BRAF p.N581I (22.65%), KMT2C p.N2587*fs*1 (19.82%), EphA5 p.R896H (12.65%), B2M p.M1T (4.47%), and MYC genes (copy number gains, n=3.61). The mutant genes and mutation abundance examined from the ctDNA obtained from the peripheral blood were TP53 p.E298* (27.58%), APC p.Q1429* (45.95%), RAF1 p.R391W (25.58%), BRAF p.N581I (22.15%), KMT2C p.N2587*fs*1 (18.13%), and STK11 (copy number losses, n=1.1), respectively. The NRAS/KRAS genes were wild type. Detailed sequencing results are shown in **Supplementary Table S1**. The PD-L1 expression was 2% (CPS, **Figure 3C**). Therefore, cetuximab (800 mg) was added to the treatment regimen. After four cycles, since the enlargement of liver metastases, radiological evaluation demonstrated PD (**Figure 3D**) according to the Response Evaluation Criteria in Solid Tumors (RECIST) version 1.1. A second-line of chemotherapy with FOLFIRI plus bevacizumab (irinotecan 240 mg day 1, leucovorin 500 mg day 1, 5-FU 500 mg IV bolus on day 1, then 3,000 mg 46 h IV continuous infusion, every 2 weeks, bevacizumab 300 mg day 1, every 2 weeks) were followed in April 2021. At the time of evaluation after five sessions, magnetic resonance imaging (MRI) showed new metastatic lesions in the skull (**Figure 3E**).

**Figure 4:**
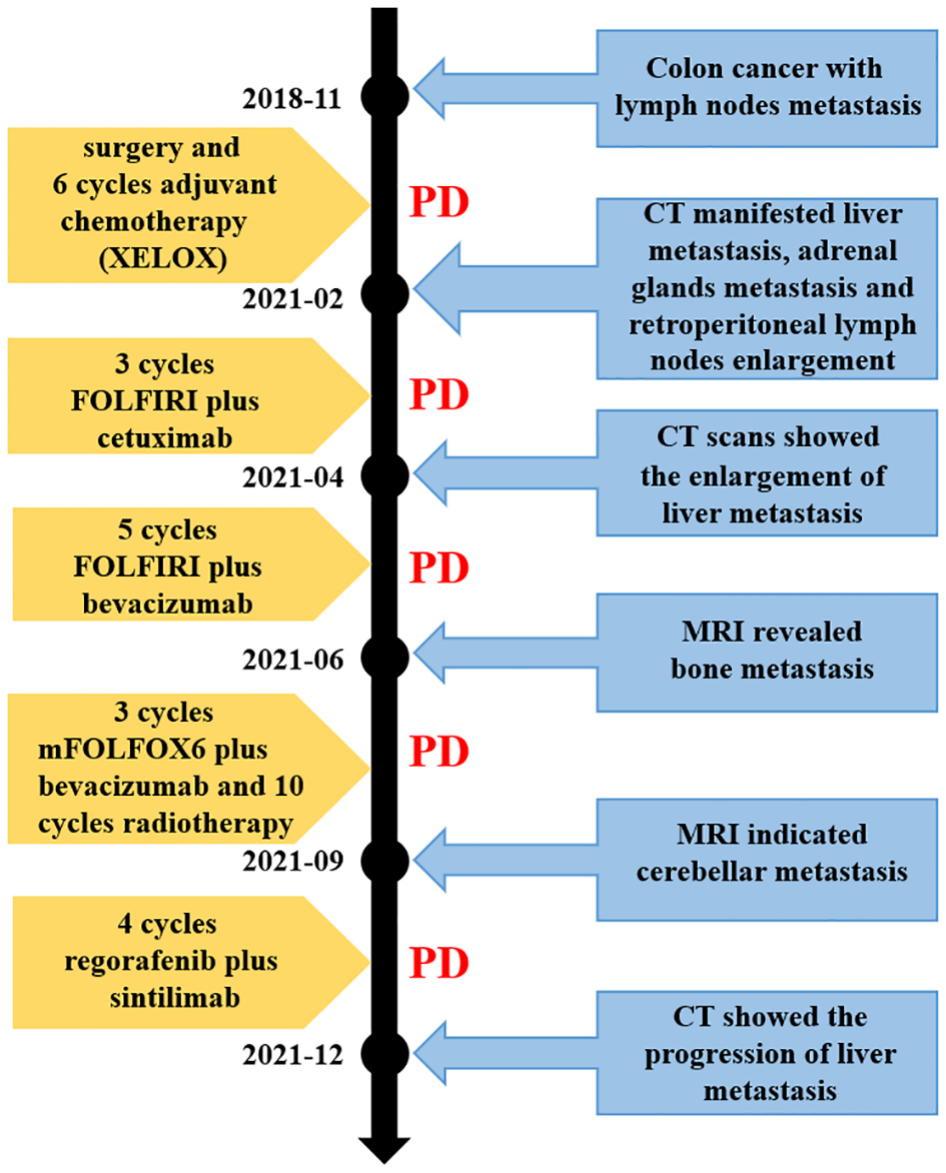
Timeline of the disease evolution. XELOX, chemotherapy regimen containing capecitabine and oxaliplatin; FOLFIRI, chemotherapy regimen containing irinotecan, leucovorin and 5-FU; mFOLFOX6, chemotherapy regimen containing oxaliplatin, leucovorin and 5-FU. CT, computed tomography; MRI, magnetic resonance imaging; PD, progressive disease.

Since the treatment was unsuccessful, chemotherapy was changed to mFOLFOX6 in June 2021 (oxaliplatin 120 mg day 1, leucovorin 500 mg day 1, 5-FU 500 mg IV bolus on day 1, then 3,000 mg 46 h IV continuous infusion, every 2 weeks) and continued the original dose of bevacizumab treatment. During the period, the patient received a short course of palliative radiation (30 Gy over 10 fractions) and bisphosphonate (zoledronic acid) due to bone metastasis. The adverse reactions of increased cortisol and hypertension appeared, but both improved after the administration of the drugs. Two months later, liver metastasis did not seem to increase significantly, while the follow-up MRI imaging presented cerebellar metastatic lesions (**Figures 3F, G**). Based on these results, she was then treated with regorafenib (80 mg) plus sintilimab (an anti-PD1 Ab, 200 mg) for four cycles. The patient received her last treatment on 25 November 2021, then suspended treatment due to hepatic dysfunction. On 21 December 2021, the patient had an abdominal CT performed at our hospital for the last time, and the liver lesion was enlarged again (**Figure 3H**). After that, she never returned to the hospital. **Figure 4** shows the treatment administration of the patient.

## Discussion

While the percentage of the BRAF V600 mutations in metastatic CRC (mCRC) range from 5% to 9%, non-V600 mutations at an estimated only 1–2% (6). BRAF mutations can be classified into three categories based on the signaling mechanism and kinase activity. Mutations occurring at BRAF V600 such as p.V600E/K/D/R/M cause the expression of RAS-independent active proteins, which are grouped as class 1 BRAF mutation (7). Class 2 BRAF mutations such as p.K601E/N/Q, p.L597Q/R/S, p.G469A/R/V, and p.G464A/R/V confer intermediate to high BRAF activity, which are dimerization dependent (8). Both class 1 and class 2 BRAF mutations become largely independent on their upstream regulator, RAS GTPase, for growth and proliferation in cancer (9). Class 3 BRAF mutations such as p.G466A/E/R/V, p.N581I/K/S/Y, p.D594A/E/G/H/N/V, and p.G596R/C significantly decrease BRAF activity, which are relying on the RAS signaling. They bind more tightly than wild-type BRAF to RAS-GTP, leading to an increased ERK signaling (10).

Compared with V600 mutations, the non-V600 mutations most often occur in men, younger ages, well-differentiated, node-negative, the left colon, and rarely present microsatellite instability-high/mismatch repair deficiency (MSI-H/dMMR) (11, 12). A diverse prognosis between the BRAF V600 and non-V600 mutant mCRC patients was observed, with a substantially longer mediate OS (mOS) of 60.7 months in the BRAF non-V600 mutated patients, 11.4 months in V600 mutated, and 43.0 months of BRAF wild-type patients. The survival difference indicates less aggressive behaviors of the BRAF non-V600 mutant mCRC (13). It has been reported that mOS in the BRAF-mutated class 1–3, and BRAF-wild-type was 21.0, 23.4, 44.5, and 42.2 months, respectively (14). Class 1 and 2 BRAF mutant mCRC patients experience poorer prognosis compared to those with class 3 patients. Class 3 BRAF mutations, like BRAF p.N581I, tend to have better prognosis than BRAF wild-type. Nevertheless, the case that we present here had lymph nodes metastasis at the initial diagnosis and sequentially massive metastasis including the bone. The OS was approximately 10.0 months, which is much shorter than the previous reports.

Due to the current findings that class 3 mutations of CRC have an overall indolent course, it is conceivable that patients with class 3 BRAF mutations may not require the aggressive chemotherapeutic regimens, which are benefits to V600E mutant CRC (10). Current RAF inhibitors are unlikely to suppress ERK signaling in the class 2 or 3 mutant-driven CRC, since RAF inhibitors effectively inhibit mutant monomers, but not dimmers (15). Yaeger et al. conducted a retrospective multicenter study involving 28 patients of class 3 BRAF mutations and observed the efficacy of anti-EGFR treatment (16). Seven of nine mCRC patients with class 3 BRAF mutants respond to treatment containing an EGFR antibody in the first- or second-line setting, objective response rate (ORR) 78%. In the third or later line, 37% patients with class 3 BRAF mutant mCRC responded. A patient with the BRAF p.N581I mutation received in irinotecan and cetuximab in the first-line was evaluated as stable disease (SD). The progression- free survival (PFS) exceeded 10.3 months. A patient with the BRAF p.N581S mutation reached partial response (PR) after being treated with FOLFIRI and panitumumab in the first-line therapy, and the PFS approximate reached 16.0 months. Similarly, one BRAF p.N581T mutated patient received FOLFOX combined with panitumumab in the first-line was evaluated as PR. Therefore, these data indicate chemotherapy combined with anti-EGFR seems to achieve good effects on these mutated populations, especially in the first-line treatment. However, the case that we report here is not consistent with previous findings. Her disease progressed after using cetuximab in combination with FOLFIRI in the first-line therapy. The disease course progress rapidly, the PFS was not more than 3.0 months.

As a matter of fact, class 3 BRAF alterations require a second hit upstream, which can be a RAS mutation or EGFR activation (17). In CRC and lung adenocarcinoma, the predominant mechanism of RAS activation upstream of class 3 BRAF mutants mainly from receptor tyrosine kinase (RTK) activation, while in melanoma are RAS or NF1 alterations (18). Therefore, blocking upstream RAS signaling is a potential treatment strategy for CRC with class 3 BRAF mutations. Based on the class 3 BRAF mutations depend on upstream EGFR signaling, our patient is supposed to respond to anti-EGFR treatment. The reason for EGFR inhibitors’ resistance to BRAF p.N581I mutation and other class 3 BRAF mutations, to a large extent due to these BRAF mutations being RAS dependent, every upstream signal activates RAS can lead to these mutants being intrinsically resistant to anti-EGFR (19).

After failure in the first-line treatment, the patient emerged multi-drug resistance in the subsequent treatment. In the second-line treatment, we chose bevacizumab, which can target vascular endothelial growth factor receptor (VEGFR), combined with chemotherapy mFOLFOX6 (20). However, the patient did not benefit from these treatments, and the PFS was still <3.0 months. According to the REGONIVO study, which achieved an ORR of 33% and a median PFS of 7.9 months when treating with nivolumab and regorafenib in MSS metastatic colorectal cancer patients who had progression after standard chemotherapy (21), we have explored sintilimab plus regorafenib as treatment options in the later-line therapy. However, even regorafenib, which targets multiple targets such as BRAF, VEGFR1-3, v-kit Hardy– Zuckerman 4 feline sarcoma viral oncogene homolog (KIT), and platelet-derived growth factor receptor beta (PDGFRB) (22), had not been able to control our patients’ disease and failed to enhance the effect of immunotherapy. From the perspective of the total survival period, OS was only approximately 10.0 months. This poor survival suggested that the malignant degree of the BRAF p.N581I mutation was very high.

EphA5 belongs to the family of RTKs and is involved in the RAS-MAPK pathway (23). The EphA5 mutation probably activated RAS leading to anti-EGFR resistance. NGS analysis of the patient revealed the EphA5 p.R896H mutation in FFPE, which likely increased the level of RAS-GTP, amplified the MAPK pathway signaling, and was involved in the multi-resistance. This pathological type of mucinous adenocarcinoma of the patient, which has been reported in diminishing benefit to anti-EGFR therapy (24), also led to the failure of cetuximab treatment. The STK11 copy number loss was obtained from the ctDNA sample. In the background of the BRAF p.N581I mutation, it may activate the PI3K pathway, as it has been found in the BRAF p.V600E mutant melanoma cells (25).

The combination of MEK/ERK inhibitors in the treatment could be considered. One patient with the BRAF p.N581S mutation in lung cancer was resistant to anti-EGFR treatment; PR was maintained for more than 33 months after changing treatment to dabrafenib plus trametinib (26). In addition, a phase I multicenter trial demonstrated that the tumor volume of BRAF non-V600 mutant patients was observed to be reduced after taking ERK1/2 inhibitor Ulixertinib (BVD- 523) (27). This trial had shown early evidence of clinical activity in NRAS- and BRAF V600- and non-V600-mutant malignant solid tumor. However, more verification is needed in the future.

In conclusion, the case report here presents a rare mCRC carrying the BRAF p.N581I mutant. The patient was resistant to the anti-EGFR treatment, anti-VEGFR treatment, and conventional chemotherapy, which was inconsistent with previous treatment cognition about class 3 BRAF mutations. The rapid progression and massive metastases indicate that class 3 BRAF mutations cannot be simply considered into a type with a good prognosis, and a comprehensive genetic profiling is essential to cancer treatment. Mutations at different sites of BRAF may influence the protein functions differently, and understanding the functional changes associated with a specific mutation is critical in choosing appropriate treatment strategies. As the NGS analysis is generally used in clinical practice, more and more rare mutations will be detected. The clinical experience that we shared hopefully will be beneficial to similar patients and helpful to the individual treatment decision. The shortcoming in the case is that we did not perform real-time NGS analysis, which led to the unclear ctDNA information of the patient with PD. It is possible that there are other mutant genes resulting in subsequent drug resistance and driving the distant metastasis of the bone and cerebellum.

## Data availability statement

The original contributions presented in the study are included in the article/**Supplementary Material**. Further inquiries can be directed to the corresponding author.

## Ethics statement

Written informed consent was obtained from the patient's family for the publication of this case report.

## Author contributions

XW collected the clinical data, composed the manuscript, and drew the figures. CZ performed the literature research. YG was responsible for supervising the report. YW participated in patient treatment and provided comments. FG designed the clinical treatment for the patient and critically reviewed the report. All authors contributed to the manuscript and approved the submitted version.
