# Supplementary material for: Multidrug resistance in the standardized treatment of colon cancer harboring a rare fibrosarcoma B-type (BRAF) p.N581I mutation: a case report
Source: Front Oncol. 2023 Jul 14;13:1175693. doi: 10.3389/fonc.2023.1175693 (PMC10380923; doi:10.3389/fonc.2023.1175693)
Supplement: Supplementary file 1 [file Table_1.docx]

**Supplementary Table legends**

**Supplementary Table 1** The results of sequence analyses of paraffin sections and blood cells.

| Gene | Mutation site | Mutation abundance  (Paraffin sections) | Mutation abundance  (ctDNA) |
| --- | --- | --- | --- |
| TP53 | c.892G>T  p.E298* | 37.52% | 27.58% |
| APC | c.4285C>T p.Q1429* | 33.35% | 45.95% |
| RAF1 | c.1171A>T p.R391W | 23.56% | 25.58% |
| BRAF | c.1742A>T p.N581I | 22.65% | 22.15% |
| KMT2C | c.7757_7758insA p.N2587*fs*1 | 19.82% | 18.13% |
| EPHA5 | c.2687G>A p.R896H | 12.65% |  |
| B2M | c.2T>C  p.M1T | 4.47% |  |
| MYC | copy number gains | n=3.61 |  |
| STK11 | copy number losses |  | n=1.1 |
